# Deconstructing design thinking as a tool for the implementation of a population health initiative

**DOI:** 10.1186/s12961-022-00892-5

**Published:** 2022-08-19

**Authors:** Caitlin Jarrett, Yara C. Baxter, Johannes Boch, Conrado Carrasco, Daniel Cobos Muñoz, Karina Mauro Dib, Lara Pessoa, Jasmina Saric, Mariana Silveira, Peter Steinmann

**Affiliations:** 1 Swiss Tropical and Public Health Institute, Allschwil, Switzerland; 2 University of Basel, Basel, Switzerland; 3 Novartis Foundation, Basel, Switzerland; 4 Universidade Federal do ABC, São Bernardo do Campo, Brazil; 5 Secretaria Municipal da Saúde, São Paulo, Brazil; 6 Universidade Estadual do Rio de Janeiro, Rio de Janeiro, Brazil; 7 Instituto Tellus, São Paulo, Brazil

**Keywords:** Design thinking, Process evaluation, Implementation science, Innovation, Health systems, Global health initiatives, Hypertension, Cardiovascular disease

## Abstract

**Background:**

The translation of evidence-based practices and rapid uptake of innovations into global health practice is challenging. Design thinking is a consultative process involving multiple stakeholders and has been identified as a promising solution to create and apply implementation strategies in complex environments like health systems.

**Methods:**

We conducted a process evaluation of a real-world example, namely an initiative to innovate hypertension screening, diagnosis and care in São Paulo, Brazil. The parameters of the evaluation were informed by a specification rubric and categorization system, recommended for the investigation of implementation strategies, and the double-diamond conceptual framework to describe and examine the strategic architecture and nature of the design thinking approach, with particular emphasis on identifying potential areas of “value-add” particular to the approach. The retrospective evaluation was performed by an independent partner who had not been involved in the setting up and implementation of the design thinking process.

**Results:**

The evaluation unveiled a dense catalogue of strategically driven, mostly theoretically based, activities involving all identified health system stakeholders including patients. Narrative reconstruction illuminated the systematic and coherent nature of this approach, with different resulting actions progressively accounting for all relevant layers of the health system to engineer a broad selection of specific implementation solutions. The relevance of the identified features and the mechanics used to promote more successful implementation practices was manifested in several distinct ways: design thinking offered a clear direction on which innovations really mattered and when, as well as several new dimensions for consideration in the development of an innovation mindset amongst stakeholders. It thereby promoted relationship quality in terms of familiarity and trust, and commitment to evidence-based enquiry and action. Design thinking was also able to navigate the territory between the need for intervention “fidelity” versus “adaptation” and provide the operational know-how to face familiar implementation hurdles. Lastly, it brought a new kind of skill set to the public health stakeholders that incorporated diplomacy, multidisciplinary approaches and management sciences—skills that are considered necessary but not yet widely taught as part of public health training.

**Conclusions:**

Design thinking is a sound and viable tool to use as part of an implementation strategy for engaging with health system stakeholders and successfully translating evidence-based practices and new innovations into routine practice, thereby addressing an important knowledge—practice gap and, more broadly, contributing to the strategic repertoire available to implementation science.

**Supplementary Information:**

The online version contains supplementary material available at 10.1186/s12961-022-00892-5.

## Background

Evidence-based public health decision-making requires knowledge not only about effective interventions but also about strategies for their successful implementation, to achieve the intended public health goals. However, in global health there is a poor history of translating proven evidence-based practices and promising health system interventions into real-world settings [1, 2]—often referred to as the know–do gap [3, 4]. A key challenge is the translation of successful pilot approaches into routine implementation without increased monitoring, resulting in an effectiveness decay over time. Maintaining the theoretical efficacy of tools and interventions under routine conditions requires listening to, and addressing the needs of, frontline workers—those who deliver the intervention—and listening to patients to understand factors governing health-seeking behaviour and treatment adherence [5]. Historically, whilst well-meaning, implementation research contributed to this issue empirically at the expense of giving attention to the theoretical underpinnings of implementation [6]. Now there is wide recognition of the need to establish the theoretical bases of implementation strategies, and a proliferation of theories, models and frameworks have emerged [7]. Whilst these tools identify diverse barriers to, and enablers of, implementation, many lack a clear specification of determinants associated with implementation success [7]. As such, public health decision-makers, programme planners and practitioners are left wanting for better “how-to” guidance to integrate and adapt best practices and recommendations into local health systems in a way that is synchronous and truly reflects local needs. Similarly, health system research has often not been designed in the frame of a systematic process but has rather been driven by theoretical considerations and practical needs [8, 9].

Design thinking represents a potential implementation strategy to bridge these theoretical and technical gaps and advance global best practice for healthcare delivery. However, as a relatively new approach in public health, and as with other systems approaches [10, 11], evidence is needed to substantiate the potential value of design thinking in a health systems context. The purpose of this paper is to present and examine design thinking as an approach to adapt a global population health initiative for cardiovascular health to the specific conditions of São Paulo, Brazil. We also assessed its potential value as an implementation strategy to achieve implementation success and sustainable practice transformation in global health services.

### Health system complexity and rapidly changing demands

Over the past two decades, implementation challenges have been compounded by shifting expectations of health systems. Specifically, systems need to adapt in order to respond adequately to rapidly changing health challenges (e.g. urbanization, increasing burden of noncommunicable diseases [NCDs]), and to deliver on calls for a more fundamental paradigm shift in the way health services are funded, managed and delivered [12]. Overall, there is an emphasis on maximizing social value including through prevention whilst producing better health outcomes [13]. These expectations should be met whilst operating in an already pressured environment of increasing healthcare costs and decreasing per capita resources [1]. The challenge is even more acute for health systems in resource-constrained settings [12].

Clearly, this is no easy transition. Health systems are complex adaptive systems comprising highly heterogeneous groups of actors (i.e. different types of health providers, managers, policy-makers, patients, regulators, funders) intervening at multiple levels through a variety of services and functions [14]. In contrast to the more traditional disciplines within public health, like epidemiology, interactions of system components are typically complex and nonlinear, so they are also not easily controlled or predicted [14]. They act in parallel and constantly react to what other “agents” are doing, which in turn influences behaviour and the network as a whole [15]. Under such conditions, problems become inherently complex or “wicked”—a “class of social system problems which are ill-formulated, where the information is confusing, where there are many clients and decision-makers with conflicting values, and where ramifications in the whole system are thoroughly confusing” [16].

In recognition of this challenge, and as the stakes grow ever higher, opinions about how to engage with health challenges within a systems context are shifting. Specifically, there is increasing recognition of the contextual limitations [14] of continuing to use linear biomedical approaches based on linear predictions and reductionist thinking [17–19] to solve system problems. There is also a rapidly growing desire and demand for more innovative [1], holistic and nonlinear methods for global health system improvement efforts [20, 21]. Achieving national and global health goals requires dynamic approaches that can appreciate the multifaceted and interconnected relationships amongst health system components and varied stakeholder perspectives [22]. Support is also required for more pragmatic ways of working to enable successful translation and sustainable implementation of relevant interventions [4].

### Systems approaches and design thinking

Systems approaches hold several attractions. One, they enable a more integrated biopsychosocial way of thinking about health [23]. Two, they are specifically designed to address complex problems [10]. And three, they allow for meaningful participatory research that brings stakeholders—patients, health workers, managers and politicians—together. In this way, the value of the population as partner and co-participant in discussions addressing shared health problems is made paramount—a necessary commitment to the practice of public health [17] and in alignment with current strategic directions to develop more integrated and people-centred health services [12].

Design thinking is a systems thinking approach that has been used successfully for several decades by diverse organizations across both the public and private sectors [24] to facilitate the evidence-to-practice leap and support organizational change. There are different models of design thinking [25], but in general the approach can be described as a social technology [26], offering a structured and defined creative problem-solving method that emphasizes engagement, dialogue and learning in order to enable and accelerate successful innovation. Moreover, it operates well in contexts characterized by uncertain environments and complex problems [27] and can embrace the uniqueness of the local context in terms of social, political and cultural idiosyncrasies [14]. In terms of expected benefits, Liedtka [26] explains that design thinking supports innovation because it is able to (i) produce superior solutions based on well-founded problem definitions, (ii) reduce the risk of failure upon implementation due to the continuous stakeholder engagement and feedback, and (iii) deliver employee or end-user buy-in. In combination, these features make design thinking a promising innovation tool and implementation strategy to use in health system strengthening.

### An evidence-building opportunity: design thinking and the Better Hearts Better Cities initiative

Heart disease is a major cause of morbidity and mortality worldwide, with highest prevalence in urban populations and hypertension as one of its main risk factors [28, 29]. In 2017, the Novartis Foundation launched the Better Hearts Better Cities urban population health initiative—hereafter “the initiative”—aimed at improving cardiovascular health in high-prevalence urban communities in three low- and middle-income countries, focusing on hypertension as a key risk factor and designed to build a model that can be replicated across settings and for other cardiovascular risk factors [30]. The initiative was implemented across Dakar (Senegal), São Paulo (Brazil) and Ulaanbaatar (Mongolia). Engagement in Brazil was initiated in late 2016 and reached the megacity of São Paulo in April 2017, where the initiative was locally named Cuidando do Seu Coração and became operational in 2018, with Instituto Tellus as implementation partner. It sought to develop and implement an innovative programme to improve the healthcare system on different levels, following the CARDIO framework, shorthand for quality of Care, early Access, policy Reform, Data and digital technology, Intersectoral collaboration, and local Ownership [28].

The decision to engage with Brazil was based on several criteria: first, the characteristics of the healthcare system—Brazil has a public health system [31] that recognizes health as a universal constitutional right, provides access to medicines [32] and vests the responsibility to execute primary care delivery at the municipal level. Second, the burden of hypertension in São Paulo is high and is increasing [33], and the health system faces burgeoning pressures in the face of urbanization, a double disease burden where NCDs overtake communicable diseases, and political interference. It is understood that there remains ample opportunity to make greater use of known evidence-based practices for reducing hypertension in resource-constrained settings [29], despite documented improvements in hypertension diagnosis and management across several geographies [34]. And third, the government of the municipality of São Paulo took leadership in the initiative and approved a multisectoral collaborative approach. As such, the initiative came at an opportune time and carried the potential for positive change. Design thinking was identified as the implementation tool of choice to translate the CARDIO framework into local action. 

### Study aims and objectives

In this study, we seek to answer the question: *Does design thinking carry value as a strategy to bridge the evidence–practice gap in health systems?* To do this, we aimed to set out what design thinking in a health systems context looks like in terms of process and nature, and define how it is expected to function to support implementation. Accordingly, our objectives were to:

1. retrospectively *identify, describe* and comprehensively *specify* the activities undertaken in the adaptation phase of the initiative rollout in the Itaquera district of São Paulo, Brazil;
2. critically review the findings, with particular attention to *implementation strategies* used and expected implementation *outcomes* to delineate dimensions of potential value.

## Methods

### Capturing the design thinking process: data sources and data collection

An in-depth retrospective list of primary activities undertaken to establish and run the design thinking process was generated by Swiss TPH, an independent evaluation partner not involved in the decision to deploy nor the implementation of the design thinking process. Data were collected through a review of project documents and interviews with the core implementation team from Novartis Foundation and Instituto Tellus. This activity list covered activities that took place preceding the selection of São Paulo as a participating city, and the subsequent project work undertaken during the pilot phase. The pilot took place in Itaquera district and incorporated six pilot primary care clinics representing the different administrative models for the local delivery of primary care, namely under direct supervision of the municipal health secretariat or managed by the social organization Santa Marcelina. Key evaluation steps included building familiarity with the locally deployed design thinking approach, understanding its essential logic, and examining its strategic character to appreciate dimensions of value to inform future replication in terms of process and expected benefits (implementation outcomes). Details were captured in a custom-designed Excel table (Additional file 1: Table S1) and narrative (Additional file 2: Table S2) format.

### Analytical frameworks and data analysis

**Fig. 1:**
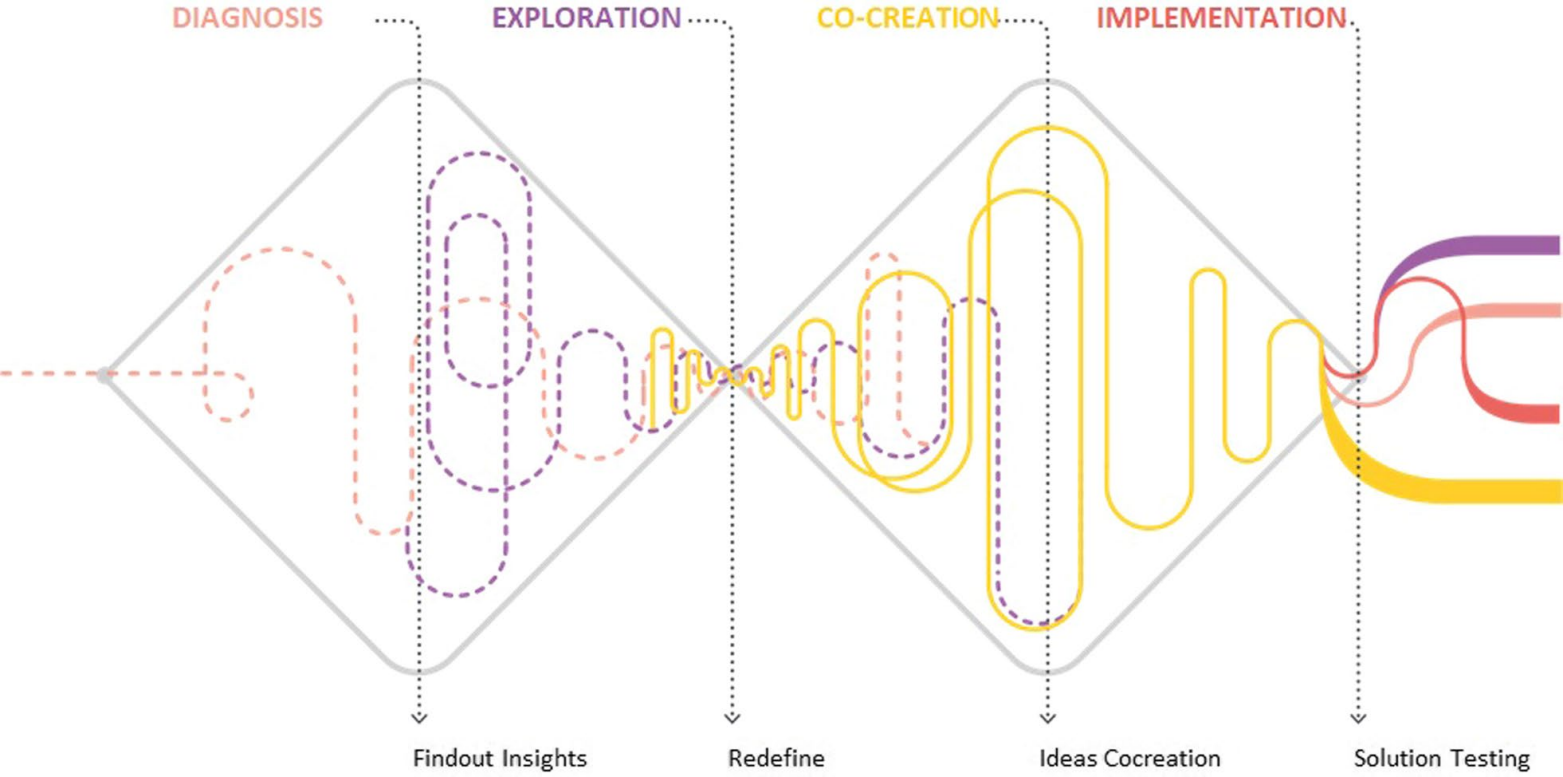
Double-diamond concept used in design thinking to visually illustrate the pattern of divergent and convergent thinking that defines the four phases of the approach: diagnosis, exploration, co-creation and implementation

There is little guidance in the literature on the best approach for evaluating implementation strategies in the context of complex, multicomponent interventions. There can be many different strategies running at the same time that are frequently overlapping [35]. Therefore, we adapted the structure of the Excel table using a combination of sources in addition to referencing the Standards for Reporting Implementation Studies (StaRI) statement [36] and minimum reporting standards indicated elsewhere [37]. The primary sources included the following:

First, the double-diamond concept [38] used in design thinking to visually illustrate the pattern of divergent and convergent thinking that defines the four traditional phases of the approach. They include diagnosis, exploration, co-creation and implementation (Fig. 1). The last phase—implementation—was initially included in the process review but later set aside because it generated a large amount of data and content that reflected a significant step-change in the process. Instead, we included an additional phase—groundwork—to capture introductory activities. All primary activities were chronologically mapped to the phase in which they began, although it is possible, and indeed expected, that the impact of many will carry influence in later phases.

Second, to align with recommendations for reporting on the use of implementation strategies with greater specificity and provisions [37], each activity was described according to Proctor et al.’s [39] specification rubric, that sets out seven domains including: the actors involved, actions undertaken, action targets, timing or temporality, dose, implementation outcomes and theoretical justification. The specification of strategies is desirable for several reasons: to support the development of an evidence base for their efficiency, cost and effectiveness; to extend the use of consistent labels and descriptions; to show any theoretical justifications for use; and lastly, to “unpack” complex interventions and provide clarity and depth for future replication [39].

Third, the actions within each primary activity were first coded to a strategic category and then linked to a related, higher-level “conceptual cluster” as proposed by Waltz et al. [40]. Conceptual clusters are referred to as strategic themes in this paper. In addition to meeting requirements around specification, this step was intended to allow for a simpler, more overarching, bird’s-eye view of the details, thereby providing a more accessible summary of activities and intent from which to consider the developmental nature (timing and sequencing) of the design thinking approach. Lastly, theoretical implementation outcomes (TIOs) were defined, and activities mapped against them.

## Findings

The list of primary activities that took place in preparation for the initiative in São Paulo is presented in Tables 1, 2, 3 and 4. The strategic themes are mapped to the activities in Additional file 3: Table S3. Implementation outcomes are identified for each activity in the right-hand part of the matrix (Additional file 1: Table S1).

**Table 1:** Groundwork phase—activities and domains for the development of the Better Hearts Better Cities initiative in São Paulo, Brazil

Process phase	#	Activity	When	Main actors	Dose/input (time required to develop/implement or activity frequency)	Target (of action; unit of analysis)	Actions (brief)	Purpose (brief)	Expectation/outcome (brief)
Groundwork	1	Targeting Brazilian cities	2016/2017: Salvador da Bahia, Rio de Janeiro, São Paulo	NF	Field visits, follow-ups. Total of approx. 3–4 months	Ministry of Health (Dept. of Primary Care and NCDs) and city health authorities	Building the case with key leadership figures	To identify a suitable city to run the BHBC initiative	Critically discuss opportunities; identify appropriate target city; obtain permission to engage
	2	First touch points	June–July 2017	NF	Concept note: 1 month field visits, virtual networking and country representatives One meeting (City Hall)	Primary care and NCD focal points in local government	Encourage São Paulo to be part of the BHBC initiative	Recognize role of local leaders, demonstrate systems perspective and understand challenges; promote partnership	Generate enthusiasm and stimulate discussion, leading to a letter of intent
	3	Field visit I: launch visit and feasibility analysis	25 Jul–4 Aug 2017	NF	11 days	Ecosystem stakeholders (hospitals, academics, primary care providers, politicians)	Extensive stakeholder mapping and situation analysis	Introduce initiative and identify support; start to frame implementation format	Establish working groups; confirm buy-in; identify possible champions; co-create target activities, locations and groups
	4	Stakeholder meetings and workshops	Between 2017 and mid-2018	NF	2017/2018: approx. every 3 months	City Hall	Keeping stakeholders up to date and ready for new insights	To maintain relationships and create a platform to showcase value-add in the face of changing political contexts	Build relationships and establish strong engagement at the local operational level
	5	Field visit II: Stakeholder consolidation and alignment	14–29 Sept 2017	NF	2 weeks	Secretary of Health; potential local partners; primary healthcare providers; situational assessment partner	Stakeholder engagement; understanding context	To share decision-making, remain sensitive and responsive to needs, promote compatibility across the initiative	Consolidate high-level commitment to the initiative and working partnerships; check in on current activities
	6	Field visit III (A): tender process and preparatory meetings	Full tender process: Nov 2017–Jan 2018 Field visit: 6–15 Dec 2017	NF	9 days Several preparatory meetings 1 workshop (district officials, Santa Marcelina and Tellus) (see below)	Public agents responsible for primary care and NCDs in local government	Selection of implementation partner and governance committees	Formalize commitment to the initiative, including shared decision-making	Selection of implementation partner and sites; confirmation of committee members; local government acceptance of preconditions
	7	Field visit III (B): workshop with technical leads	14 Dec 2017	NF with support from Tellus team	1 half-day (4 hours)	Representatives with coordinating, strategic, technical and medical roles	Kick-off workshop with technical leads	Open forum to discuss predefined questions and encourage further inquiry to address doubts and concerns	Introducing the initiative and informing strategic action; exchange between actors to promote trust and positive engagement
	8	Project kick-off	23 Feb 2018	Tellus team	Workshop preparation: 10 days Workshop: 1 day	– East zone coordination – Itaquera supervisors – Social organization partners – Government representatives	Project kick-off with Tellus	Build relationships amongst stakeholders, confirm next steps	Validation of schedule and process; strengthening of relationships; ownership

*BHBC* Better Hearts Better Cities, *NCD* noncommunicable disease, *NF* Novartis Foundation

**Table 2:** Diagnosis phase—activities and domains for the development of the Better Hearts Better Cities initiative in São Paulo, Brazil

Process phase	No.	Activity	When	Main actors	Dose/input (time required to develop/implement or activity frequency)	Target (of action; unit of analysis)	Actions (brief)	Purpose (brief)	Expectation/outcome (brief)
Diagnosis	9	Desk research	Feb 2018	Tellus team	1 month	– HTN patients – Other cities involved – Context of São Paulo and Itaquera	Disease, BHBC initiative and local context	Familiarization with disease, content and context	Comprehensive learning about target group and health systems Identification and alignment with priority needs
	10	CSD Matrix development	Feb 2018	Tellus team	1 month	– Design team (visual summary/thinking tool) – Other initiative stakeholders	Consolidation of research findings	Visual summary of evidence to guide next steps	Creation of a visual summary of evidence as a foundation for learning, critical thinking and research action
	11	Meetings with east zone coordination	27 Feb 2018	Tellus team	20 (approximately)	– East zone coordinator – Itaquera district – Santa Marcelina social organization	Stakeholder validation of research tools and schedule	Relationship-building, shared decision-making and needs alignment	Confirmation from stakeholders about the next stage, focal areas and timeline Strengthening of stakeholder and team relationships
	12	Interviews and co-creation sessions in primary care clinics	26 Feb–26 Mar 2018	Tellus team	1 month field interviews: 80 interviews (patients, supervisors, health professionals and support staff) 10 co-creation sessions with patients and management council	– Territory supervisors and healthcare workers and administrative staff – Itaquera community – Health unit management councils	Primary data collection–Interviews and co-creation sessions	Build knowledge, frame and reframe to achieve target focus	Understanding of engagement and user needs for validation of hypotheses and clarity on design work Research and inclusion of additional stakeholders to build connection
	13	Systematization, initiative status update and live event	Mar 2018	Tellus team	Systematization process: 10 days Status updates: five meetings (individual meetings with partners)	NF, City Hall, American Heart Association	Frame findings to establish innovation framework, stakeholder involvement and opportunities	Converge, review and distil knowledge	Confirmation of key findings and subsequent definition of next steps based on evidence review Regular face-to-face meetings

*BHBC* Better Hearts Better Cities, *CSD* certainties, suppositions and doubts, *HTN* hypertension/hypertensive, *NF* Novartis Foundation

**Table 3:** Exploration phase—activities and domains for the development of the Better Hearts Better Cities initiative in São Paulo, Brazil

Process phase	No.	Activity	When	Main actors	Dose/input (time required to develop/implement or activity frequency)	Target (of action; unit of analysis)	Actions (brief)	Purpose (brief)	Expectation/outcome (brief)
Exploration	14	Observational research: A day in the life of CHAs	Mar 2018	Tellus team	100 visits were shadowed	– CHAs (from two pilot sites)	Observational research	Build empathy with key stakeholders	To extend user-focused research and insights generation Direct engagement with stakeholders to build empathy and promote trust
	15	Field research and interviews	Mar–Apr 2018	Tellus team	6 days in total—1 day per pilot health unit	– Itaquera community – HTN patients – Pilot health units and staff	Field research—system structure and dynamics	Build a dynamic picture of the structural environment influencing the aetiology of HTN	To understand HTN links between establishments within the Itaquera territory and community behaviour
	16	Observational participation in activity groups at primary care clinics	Apr 2018	Tellus team	One activity group observed for 10 hours	HTN patients attending activity Primary care workers (health technicians, CHA, managers)	Observational research—HTN services in primary care	Understanding current patient services and engagement	To understand patient services and health behaviours To build relationships with pilot sites and patient communities
	17	Co-creative meetings with managing councils of each clinic	Apr 2018	Tellus team	6 meetings with co-creation session; 1 hour per meeting	Managing councils (healthcare professionals, health system managers and community representatives/clinic users from each UBS)	Co-creation sessions	Check learnings about system and processes; deeper examination of needs and ideas; promote interest for sustainability	To understand system processes and develop and enhance relationships
	18	Establishing baselines: questionnaires and desk research	Apr 2018	Tellus team	6 days; 8 hours a day	– Primary care workers – Clinic managers	Hard and comparative data at different levels of the health system	Establish baseline for evaluation; extend evidence base to direct action; build relationships	To establish a set of indicators for subsequent evaluation To characterize HTN care and treatment To strengthen relationships
	19	Evidence review, co-creative sessions—patient adherence and health information	Apr 2018	Tellus team	Desk research (10 days) 6 interviews (pharmacists) 6 co-creation sessions (five patients per session)	HTN patients Local population Health information system (secretary, coordination, supervisors, UBS, patient)	Review of evidence on patient adherence, local experiences, health information system	Identify system levers to support patient adherence	To promote evidence-informed (global–local) thinking to support the design of desirable, feasible and acceptable innovations
	20	Clinical guideline review and curriculum review	Apr 2018	Tellus team	10 meetings with co-creation sessions 18 questionnaires 18 interviews	NF Medical Society Itaquera and Santa Marcelina supervision	Assess clinical guidelines use, professional training and user journeys	To develop guidelines based on understanding of system functioning and system actor perspectives; augment designer–stakeholder relationships	To reach an informed position to redesign guidelines to promote improved execution To show a deep appreciation for user experiences To better understand key challenges and needs
	21	Checkpoints	Mar–Apr 2018	Tellus team	2 meetings each month (with 1 week of preparation for each meeting)	Itaquera supervisor and supervision managers	Recurring face-to-face meetings with Itaquera technical supervisory team	United project management and team-building	To clarify specific doubts about management and accelerate the exploration process
	22	User journey formulation	Apr 2018	Tellus team	80 hours	Itaquera HTN ecosystem—people: healthcare workers, doctors, CHAs, pharmacists, patients and managers	Assemble data into visual summary—user journeys	Visual depictions of user experiences as evidence summaries (convergence) and workshop tool to guide co-creation phase (divergence)	To create journey maps for each target actor to inform design thinking
	23	Itaquera map	Apr 2018	Tellus team	40 hours	Itaquera HTN ecosystem—structure: demographic profile, location of UBS and coverage area, public and private leisure and consumer facilities	Assemble data into visual summary—territorial map	A visual guide to the structural layout of the health ecosystem to identify key targets and change opportunities	To understand the behavioural profile of users based on what services (consumer, health and leisure) are available in the territory
	24	Personas	Apr 2018	Tellus team	40 hours	Itaquera community members at risk of, or already living with, HTN	Evidence-based creation of target “personas” for HTN	User-friendly evidence summary, educational and co-creation tool	To identify target groups with challenges and behaviours as a tool for informed action in workshops
	25	Stakeholder map	Apr 2018	Tellus team	80 hours	Itaquera HTN ecosystem (people and environment)	Visualization of spheres of influence across health ecosystem	User-friendly visual summary and co-creation tool	To present the whole health ecosystem in terms of influential actors to be used as a tool for informed action in workshops
	26	Characterization of health system and living environment	Apr 2018	Tellus team	80 hours	Itaquera HTN ecosystem (infrastructure and services)	Visual profiles of pilot health units for intervention	User-friendly educational and co-creation tool	To create a setting-sensitive tool to guide and validate innovation ideas, and inform resource planning and evaluation
	27	Development of design principles for the design strategy	Apr 18	Tellus team	40 hours	Tellus team	Empathy, engagement and training as basics for the co-creation strategy	To place empathy and community engagement at the heart of design strategy	To define a set of design principles with the necessary qualities to promote the innovation and implementation process
	28	Development of guidelines for co-creation workshops	Mar 2018	Tellus team	40 hours	Future co-creation workshop participants	Guidelines for co-creation workshops based on design principles	To bring all learnings into a concrete form to educate, co-create and reference	To consolidate evidence for reference and educational materials to enable engagement and shift thinking paradigms
	29	Phase presentation to steering committee and broader group	Apr 2018	Tellus team	1 workshop split into two parts	Part 1: Steering committee Part 2: Meeting with broader group	End-of-phase presentation of project findings for validation and next steps	To complete validation rituals as necessary for the design process	To validate process and target challenges to be taken forward to co-creation workshops

*CHA* community health agent, *HTN* hypertension/hypertensive, *NF* Novartis Foundation, *UBS* Unidade Básica de Saúde

**Table 4:** Exploration phase—activities and domains for the development of the Better Hearts Better Cities initiative in São Paulo, Brazil

Process phase	No.	Activity	When	Main actors (who enacted the strategy)	Dose/input (time required to develop/implement or activity frequency)	Target (of action; unit of analysis)	Actions (brief)	Purpose (brief)	Expectation/outcome (brief)
Co-creation	30	Co-creation workshops	May 2018	Tellus team	6 workshops, approximately 2 hours each	Clinic managers (6 pilot sites) Primary healthcare workers (nurses, physicians, CHA, pharmacists and auxiliary staff)	Guided workshops to take deep dive into problems, think about solutions, and prioritize	Engaging key users and building solution champions; staying user-centred and continuing active engagement	To build understanding of purpose and develop a set of innovation skills and realistic solutions to agreed problems through shared experiential learning
	31	Co-creation workshops	May 2018	Tellus team	4 workshops, about 2 hours each	HTN patients in primary care clinics (study site units)	As above	Staying user-centred	As above
	32	Systematization of solutions	May 2018	Tellus Team	40 hours	Solutions generated in co-creation workshops – Clinic managers – Primary healthcare workers – Pharmacists – HTN patients	Collate ideas and solutions articulated in co-creation workshops	Improve structure of solutions for selection ranking	To refine the ideas based on all the information collected in the co-creation workshops
	33	Solution discussion, prioritization and selection leading to prototypes	Jun 2018	Tellus Team	5 meetings over 1 month	Final review and agreement on interventions: – NF (funders) – City Hall (local government)	Solution matrix, final decision-making, implementation plan and pilot prototypes	Transparent and comprehensive engagement with key decision-makers	To select ideas/solutions to move forward with according to established criteria
	34	Co-creation workshops	Aug 2018	Tellus team	4 workshops, approximately 4 hours each	Pharmacists from primary care clinics (all São Paulo)	As in #30	Staying user-centred and broadening solution horizon (city-wide)	To engage with a wider group of stakeholders using co-creative techniques to further adapt solutions to fit within a broader context

*CHA* community health agent, *HTN* hypertension/hypertensive, *NF* Novartis Foundation

### Groundwork (April 2017–February 2018)

This phase included the initial groundwork and strategic alignment between the high-level initiative coordinator and co-funder Novartis Foundation and leading municipal and health system decision-makers. The ground was prepared by assessing the need for an initiative, investigating challenges and opportunities with local thought leaders and academics, validating initial hypotheses against stated in-country needs, and engaging the health authorities. After obtaining this institutional green light, Novartis Foundation, the health authorities and the future implementation partner Tellus built a shared vision and common agenda about how the initiative should be developed. Overall, in terms of strategic direction, much emphasis was given to key stakeholder engagement, particularly at higher levels in municipal, district and subdistrict governments. To remain grounded in field realities, those positioned at the operational front line (i.e. primary care providers) were equally engaged to seek out potential local partners across different sectors and shape the initiative’s concept into a local programme. From the very beginning there was an awareness to proactively address sustainability, with benefits for society and the public. Relationships and responsibilities were formalized early on.

During this phase, every activity used multiple engagement strategies, which were often repeated using different techniques. For example, all except one activity (#6) contributed to the development of a needs assessment (strategic theme: use of evaluative and iterative strategies) and included elements of facilitation (provide interactive assistance). Similarly, every activity sought to contribute to building a coalition across different levels of hierarchy and responsibility, promoting network-weaving (both: develop stakeholder interrelationships). Whilst working to develop relationships, partners were encouraged to consider alternative models for the delivery of hypertension services. These efforts translated into official mandates to support change (change infrastructure) and educational meetings in the spirit of collaborative learning (train and educate stakeholders).

As regards expected benefits (i.e. TIOs), this strategic activity carried the primary intentions of establishing a working environment based on respectful exchange, mutual learning and a desire to work in partnership, where critical thinking about the possibilities could be seeded and promoted (TIOs: feasibility; acceptability; readiness for change; innovation mindset) and also spark positive affect (TIOs: acceptability; desirability; relationship quality) towards the initiative, resulting in stronger and more committed stakeholder engagement (TIOs: engagement/buy-in; sustainability of innovation).

### Diagnosis (February–March 2018)

This first official design thinking phase included a period of inquiry-based action, reflection and connection. The aim was to capture as much knowledge as possible and start to examine, understand and build group consensus about the key issues and contextual challenges for hypertension services through the local health system and how these might all be combined to dovetail with the strategic pillars of the initiative. Throughout this phase, much emphasis was given to continuously acquiring, updating and validating knowledge using an increasingly representative group of stakeholders.

To achieve this, design thinking sought to use multidisciplinary research efficiently and effectively as its primary tool for strategic action. For example, a traditional needs assessment was conducted to identify barriers and facilitators that might affect system readiness to change (strategic theme: use evaluative and iterative strategies). These findings were then combined with prior knowledge. Once consolidated, these insights were used to identify and guide additional research requirements (e.g. target subjects, interview topics) and to share acquired knowledge in local consensus meetings (adapt and tailor to context; develop stakeholder interrelationships; train and educate stakeholders).

In terms of expected benefits, it was anticipated that through this broad, inclusive and adaptive approach, a learning environment, culminating in group alignment, would be created and stakeholder engagement would be enhanced (TIOs: learning environment; acceptability; desirability [of initiative]; engagement/buy-in). This period of activity was also approached as an opportunity to positively influence implementer–stakeholder relationships and promote an innovation mindset amongst those involved (TIOs: relationship quality; innovation mindset). The rapid and iterative conversion of research findings and new information into accessible summary formats presented an element of rigor, whereby decision-making and actions were evidence-based, and additive to dimensions of sustainability (TIO: evidence-based inquiry/action; sustainability of innovation).

### Exploration (March–April 2018)

The exploration phase incorporated all layers of the health system, including patients, to build a complete picture of the local context and the way different actors interrelated with it to better understand the dynamics of hypertension care and potential trajectories of impact from any interventions. The activities during this phase centred around further multidisciplinary research that sought to represent the perspectives of key system actors and to help build empathy with actors at the health system—public user interface (e.g. shadowing community health workers, interviewing members of the public, patients and primary healthcare professionals, and actively participating in health promotion programmes).

Further, activities sought to be sensitive to the dynamics of system hierarchies and the need to foster engagement and consensus across all levels, in the belief that this supports the innovation journey. For example, individual meetings were held with the managing councils—comprising healthcare professionals, health system managers and community representatives/clinic users—of each pilot primary care clinic (*n* = 6). These meetings had a multipurpose character: to create an opportunity to share and validate new learnings from the ongoing research; to continue enquiries and foster different ways of thinking through co-creative sessions; to enable cross-system collaboration and consensus-building about the true nature of the system, processes and decision points; and to enhance relationships and understanding between the implementation team and the health system actors as well as the patients. Further efforts were made to galvanize these expectations through frequent meetings with local management teams and the creation of primarily visual evidence summaries, to bring all learnings to a point of convergence, ready to be used as education and co-creation tools in subsequent phases.

Altogether, these actions reflected an interwoven strategic approach, combining ongoing assessments of local needs, barriers, facilitators and readiness with patient feedback, local knowledge sharing and consensus discussions (strategic themes: use of evaluative and iterative strategies; engage consumers; develop stakeholder interrelationships). Moreover, research efforts were facilitated by multidisciplinary experts (provide interactive assistance) who worked at an intense pace and paid immediate attention to learnings, in an effort to promote adaptability to needs (adapt and tailor to context). Finally, the breadth of system participation and engagement reflected an emphasis on promoting network-weaving and coalition-building (develop stakeholder interrelationships).

As regards expected benefits, activities in the exploration phase were heavily directed by the guiding principle of building empathy in the belief that focusing efforts in this way would build engagement with the initiative and promote positive, and increasingly team-oriented, relations with stakeholders, including patients (TIOs: relationship quality; engagement/buy-in). It was assumed that these outcomes would be enhanced through the creation of learning environments and attention to collective, evidence-informed processes (TIOs: learning environment; evidence-based inquiry/action; innovation mindset). Independently and collectively, each of these outcomes were seen to influence the acceptability of the initiative and ultimate sustainability of related change and innovations (TIOs: acceptability; sustainability of innovation).

### Co-creation (May–June 2018)

The co-creative phase included a series of guided participatory workshops that brought together target stakeholders in hypertensive care, including patients and members of the public. The workshops were formatted to first educate participants about the initiative, using the evidence base that had been created, and then hand over the opportunity—and responsibility—for the innovation process to local experts. This approach sought to maintain user centricity and build ownership of created solutions and future action. This phase was heavily reliant on specialist design skills, but all decision-making was by stakeholder consensus.

Like all other phases, co-creation drew on many different strategies to construct an environment conducive to innovation and shared understanding, ownership and commitment. First, all participants were presented with the evidence using selected design techniques (e.g. visual evidence summaries) which were intended to allow participants to quickly understand the situation and the problems they would be working to develop solutions for, as well as shift critical thinking into a creative mode (strategic themes: train and educate stakeholders; engage consumers). Second, facilitated through specialized design techniques (e.g. idea generation, prototyping), participants entered into a rapid solution-creation process whereby they worked to tailor and adapt group ideas into feasible, desirable and acceptable solutions through verbal exchange and low-fidelity prototype testing (use evaluative and iterative strategies; provide interactive assistance; adapt and tailor to context). This highly interactive exchange was considered an opportunity to model and simulate change in a safe way, augment learning, build local consensus, promote networks, and identify innovation champions (develop stakeholder interrelationships; train and educate stakeholders; engage consumers). As a last step, technical design skills were used to facilitate decision-making on final solutions to be piloted in the implementation phase, allowing for continued adaptation and consensus-building (use evaluative and iterative strategies; provide interactive assistance; adapt and tailor to context; develop stakeholder interrelationships).

The expected benefits from these activities were to continue to promote stakeholder engagement through a learning environment in which active participation and innovative thinking were encouraged. This process was expected to elicit realistic reflections on the system and organizational readiness for change. Inclusion of the final high-level consensus meeting, during which formal commitments to the initiative in its updated form were confirmed by the participants, meant that important features for the sustainability of the innovation were met before moving into the implementation phase.

## Discussion

This study pursued two main objectives—one, to capture in detail the design thinking process pursued in the planning of a project in São Paulo; and two, to examine the strategic value and purpose of the design thinking approach. The study assumed a potential value of design thinking as an implementation strategy to reduce the know–do gap to address health system challenges related to hypertension. The use of the specification rubric proved essential to adequately describe what took place as part of the Better Hearts Better Cities programme in São Paulo. The design thinking approach is critically analysed in terms of implementation outcomes, its value as a research tool and the potential to facilitate the introduction of innovations into routine intervention packages.

### Characterization of the Better Hearts Better Cities design thinking approach

Concepts and tools to replicate design thinking processes are available [11]. For example, the process of “clarify, ideate, develop and implement” [41], the activities, environments, interactions and objects (AEIO) framework [42, 43], and journey maps or problem framing tools. The case study described here reflects several of these elements and demonstrates the viability and applicability of design thinking for a public health challenge where it might be particularly beneficial due to the needs- and user-focused approach which considers not only pain points in the patient journey but also components of health professionals, the health system, caregivers and the broader ecosystem.

In terms of process, the implementation activities remained true to design thinking's disciplinary concept, tracking to a pattern of divergent and convergent thinking and action paired with repeated cycles of consolidation and reflection to inform the deliberate placement of next steps in the implementation process. Further, the approach was liberal in its openness to method and thinking, rigorously applying multidisciplinary tools and techniques in a committed effort to promote creative and adaptive processes, but also structured and systematic, with every action serving multiple purposes that were expected to build towards successful implementation. In essence, operations were anchored to democratic and cooperative principles of engagement, and by the end of the third phase (co-creation), the approach delivered on expectations of product, namely a set of co-created solutions for pilot implementation (to be communicated separately).

### Design thinking and implementation outcomes

The specification process allowed for the delineation of expected implementation outcomes that could then be used to test whether expected benefits were achieved and assumptions held. An extra benefit of the specification process was that it highlighted the need to consider additional aspects and layers of value in at least two ways. First, it highlighted an important point of differentiation from typical implementation framework outcomes in terms of breadth and dimensionality. For example, implementation is usually seen as starting at the point of intervention adoption—and the chain of outcomes typically presented also starts at “adoption”. In contrast, the design thinking method suggests an earlier starting point and proposes a revised set of relevant outcomes alongside a rapid continuous improvement process (test-pilot-adapt-scale), specifically those that target the establishment of positive working relationships, shared ownership of the initiative, and active engagement with, and overall acceptability of, the innovation process. Further, the identification of potentially new and more nuanced outcomes (e.g. innovation mindset, evidence-based inquiry and action, and relationship quality with dimensions of familiarity and trust) provides potential candidates for inclusion in implementation frameworks and suggests that there may be use in including higher-order outcomes (e.g. resilience) that encapsulate groups of multiple, interrelated lower-order outcomes. And second, through the required articulation of purpose and justification, the specification process created a clearer narrative about the role and nature of different outcomes and showed that they carried both individual (e.g. chronological position) and collective (e.g. increasing interconnectedness as part of the evolving process) value.

Current implementation frameworks function as repositories for outcomes of interest but do not specify degrees of importance. In contrast, the design thinking approach does exactly this in that it brings its own methodological concept, design principles and theory-based practices to implementation. In particular, the approach emphasizes continuous learning, drawing stakeholders including patients into the innovation process early on as active participants and experts, anchoring to empathetically driven processes, and incrementally enabling them to acquire a more innovative mindset through visual and experiential learning. The anticipated value here is that by already being familiar and meaningfully engaged with the innovation process, stakeholders will be more receptive to changes within the system that will accompany the implementation phase and will possess the skills to manage future adaptations when the context inevitably changes. Moreover, the emphasis on learning and acquiring new skills fosters resilience, which is recognized as one of four primary values underpinning high-quality health systems [13]. Specifically, resilient systems are systems that can prepare for, and effectively respond to, crises while maintaining core functions and reorganizing if needed [44].

### Design thinking as a research tool

Whilst the design thinking approach can be considered an implementation strategy in its own right, the specification process makes it clear that it also proposes many different strategies that are operating on multiple levels and seeking diverse outcomes. Moreover, consideration of the way in which strategic activities are arranged indicates that the design thinking approach is akin to a form of implementation research and, in fact, incorporates many features that are considered best practice in the field [4]. Implementation research is defined as an integrated concept that links research and practice and uses multiple disciplines and methods while emphasizing partnerships between health system actors to create and apply knowledge in the most practical ways to improve the implementation of health policies, programmes and practices [4]. Key features—all of which were found in this case study—include an emphasis on being locally present and investing repeatedly in understanding the local context and any changes over time; working from a position of need and with a clear set of mutually agreed objectives; using multidisciplinary methods; developing and securing working partnerships across all phases with co-production and concurrent use of knowledge; and a continuous effort to nurture trusting partnerships between authorities, health system actors and patients in the face of a politicized public debate about health and the role of the state in healthcare.

### Design thinking for intervention design, adoption and adaptation

In this case study, we describe a design thinking process incorporating concentrated and repeated efforts to co-direct and co-produce the initiative with all relevant health system actors. The intention was to include many of those who would ultimately be implementing, and benefitting from, the interventions, in the design process [45]. The potential value was twofold: first, it resulted in the creation of tangible solutions that would either help to translate already existing evidence from clinical guidelines into routine practice, or suggest completely new ways of working towards priority needs and solutions ready to be piloted in the implementation phase. And second, it was expected that a co-creation process would reach context-sensitive outcomes and as a consequence, suitably prime the implementation stakeholders to accept, adopt and action innovative change as part of the implementation process, maintaining the intervention even in the event of adversity. Moreover, with all actors positioned to carry knowledge about, and affective connections with, the process, the innovation work is anticipated to be more protected from political interference. Indeed, the initiative managed to expand its activities significantly over the course of only 2 years, from 6 to currently 71 primary care clinics across two districts, despite considerable changes in the political landscape including leadership changes in relevant services.

### Design thinking and implementation discourse

Design thinking is inherently efficient, adaptive and agile. Such qualities are relevant to the fidelity–adaptation dilemma where there is continued examination of the difficulties of reconciling the importance of intervention fidelity versus intervention adaptation for successful implementation [46]. In this example we see evidence being integrated into the innovation process, with clinical guidelines informing the thinking around solutions. Where design thinking diverges from fidelity, however, is in how it handles evidence and, where necessary, makes space for novel solutions. Of note, it does not guarantee that evidence-based practices will remain immutable or that all evidence will be adequately considered. This suggest that the design thinking approach may indeed offer the technical repertoire to bridge how evidence can be integrated to optimize effectiveness.

From a global implementation practitioner perspective, there exists an “implementation bottleneck” [47]. For cardiovascular disease in low- and middle-income countries, barriers include uncertainty about the effectiveness and feasibility of interventions in different contexts; fragmented stakeholder efforts; lack of focused leadership and collaboration in line with clearly defined goals and outcomes; and insufficient financial, individual and institutional resources [48]. These reasons are familiar and recur across healthcare domains [49]. Frieden [50] sets out six components necessary for effective public health programme implementation: innovation; technical packages of evidence-based interventions; effective performance management; partnerships and coalitions with public- and private-sector organizations; accurate and timely communication to effect behaviour change and build engagement; and political commitment to resources and support for action [50]. This underlines that the primary challenge is not knowledge in itself but rather what to do with it. Design thinking incorporates all of these components but also, critically, brings knowledge about barriers and strategies together to make solutions operational.

### Design thinking and global health skill sets

A particular set of skills including diplomacy, design and communications are applied to drive the design thinking approach. For example, during the groundwork phase, an act of diplomacy played out which combined advocacy (a new way of working through multidisciplinary partnerships), negotiation (Novartis Foundation engagement with the initiative) and problem-solving with missions of reflection and problematization (e.g. needs assessment, early and repeated networking and high-level discussion) [51]. The subsequent phases employed specialist design skills, in particular the creation and use of visual tools and a thinking style that was open and integrative, that lend themselves to innovative change. The arrangement of skills is worth noting, because whilst diplomacy and communications do fall within public health competency frameworks, design does not. More critically, the extent to which key skills are recognized as being important, taught in training environments and operationalized, differs across countries. Public health training often has a narrow perspective on population health and can be restrictive in its training for nonclinical students [52].

## Limitations

Converting actions into implementation strategies using codes as defined by Waltz et al. [40] proved more cumbersome than initially anticipated. This was because, for each primary activity, there were generally numerous and diverse related actions which in turn aligned with multiple strategies. Therefore, the coding process was to some degree subjective when making the interpretive leap from action to strategy, and only one researcher performed this task.

## Conclusions

The overarching logic of design thinking for addressing health system challenges such as translating evidence-based practices is accessible but there is limited understanding of the “how” and “why” of its operation. In deconstructing the approach in the context of a real-world example, a clear set of strategic methods and expected implementation outcomes have been identified, and the nature of their interrelationships characterized. The results present new perspectives for the evolution of current implementation frameworks. We also provide critical reflection on the potential value of design thinking for overcoming broader implementation issues that are commonly debated, as well as highlighting current disciplinary deficits in public health that may be inadvertently undermining implementation efforts. Lastly, this study contributes contemporary evidence to inform a wider disciplinary discourse across health systems operations, global health practices and implementation science.

## Supplementary Information

**Additional file 1: Table S1.** Overview of the design thinking process including phases, activities, domains and implementation outcomes for the development of the Better Hearts Better Cities initiative in São Paulo, Brazil.

**Additional File 2: **Overview of the design thinking process and development of the Better Hearts Better Cities initiative in São Paulo, Brazil.

**Additional file 3: Table 3.** Action-to-strategy coding of the design thinking process to deliver the Better Hearts Better Cities initiative in São Paulo, Brazil (2016–2018).

## Data Availability

The datasets generated and/or analysed during the current study are available as supplementary files.
