# Supplementary material for: Deconstructing design thinking as a tool for the implementation of a population health initiative
Source: Health Res Policy Syst. 2022 Aug 19;20:91. doi: 10.1186/s12961-022-00892-5 (PMC9389775; doi:10.1186/s12961-022-00892-5)
Supplement: Supplementary file 2 — Additional File 2: Overview of the design thinking process and development of the Better Hearts Better Cities initiative in São Paulo, Brazil. [file 12961_2022_892_MOESM2_ESM.docx]

SUPPLEMENTARY FILE 2 – OVERVIEW OF THE DESIGN THINKING PROCESS AND DEVELOPMENT OF THE BETTER HEARTS BETTER CITIES INITIATIVE IN SÃO PAULO, BRAZIL

*Descriptive results – Primary activities and duration*

The number of **primary activities** that took place across the phases totaled 34 with the individual phase tallies as follows: Groundwork phase (n=8); Diagnosis (n=5); Exploration (n=16); and Co-creation (n=5). In terms of duration, the Groundwork phase took 10 months (April 2017 – Feb 2018 inclusive) and the remaining phases - Diagnosis (Feb – Mar 2018), Exploration (Mar – Apr 2018) and Co-creation (May – Jun 2018) - each ran for two months. The broader co-creation activities with pharmacists that developed spontaneously as part of the scope of work, took one additional month (Aug 2018). Overall, to complete the preparatory work and run the first three phases of the Design Thinking methodology took 16 months (excluding pharmacists).

**PRELIMINARY PHASE: GROUNDWORK**

|  | **Activities** | **Actions** |
| --- | --- | --- |
| **1** | **Targeting Brazilian cities** | **Building the case for BHBC with key leadership figures** |
| **2** | **First touch points** | **Networking to invite the city of São Paulo to be part of the Better Hearts Better Cities initiative** |

Early groundwork sought to build the case and place for the Better Hearts Better Cities (BHBC) urban population health initiative in a suitable city in Brazil and was led solely by the Novartis Foundation. This effort was directed by two senior team members with extensive knowledge and experience working with the Brazilian health system. Background research was conducted, and the shape of the initiative tailored to the most sense locally. At the same time, extensive networking was undertaken to connect with the right local influencers, especially at higher levels. Initial contact was directed at the Ministry of Health in Brasilia and once engaged, São Paulo was selected as the target/recipient city. Attention then turned to the public agents responsible for primary care and non-communicable diseases (NCDs) in local government (e.g., Health Secretary, São Paulo City Hall Health Dept). A concept note was authored by NF to share with key stakeholders (City Hall) and after a networking breakthrough, was eventually presented and discussed in a face-to-face meeting with the Health Secretary and their core team at City Hall (approx. 7 people).

*Purpose of actions:*

Finding the right Brazilian city had been a challenge with earlier efforts in Rio de Janeiro and Salvador da Bahia having been unsuccessful due to factors such as missing engagement from key political entities, election periods, differing priorities for primary vs tertiary care strengthening or concerns about data access. The intent therefore was to secure a target city and then initiate engagement with key figures. The pairing of the concept note with high level face-to-face meetings served several purposes: First, by preparing a comprehensive, evidence-based concept note, including a formally stated position on the primary vision – that was, to build a multidisciplinary partnership and to use a whole system approach to address current challenges around hypertension – the team aimed to demonstrate their understanding of, and genuine interest in, key local issues and a shared strategic focus. Second, clear statements as regards expectations of local political and financial commitments were meant to promote informed discussions that could progress into formal partnership with a clear understanding of responsibilities. And third, by engaging with key local leaders, the team sought to establish high-level commitment – a recognized precondition of success.

*Justification:*

Obtaining high-level input and agreement to proceed is recognized as a precondition to success in raising health systems quality (Kruk, 2018) and helps to facilitate subsequent networking to develop the initiative. Earlier efforts with other cities had been unsuccessful but after elections in 2016, a new leader took office in São Paulo City Hall in January 2017 with an agenda to drive public private partnerships. In contrast to his predecessor, he was focused on a liberal approach to accelerate delivery of public services. Under these political circumstances, it was decided that it was an opportune time to engage with São Paulo as a BHBC initiative city.

|  | **Activities** | **Actions** |
| --- | --- | --- |
| **3** | **Field Visit I: Launch visit & feasibility analysis** | **Extensive stakeholder mapping & situation analysis** |
| **4** | **Ongoing meetings and workshops with key stakeholders** | **Keeping key stakeholders up-to-date and staying tuned for new insights** |
| **5** | **Field Visit II: Stakeholder consolidation & alignment** | **Continue building key stakeholder relationships and engagement, understanding context & establishing strategically aligned partnerships** |

Continuing to be led by the NF team (n=2), the next series of activities incorporated two field visits to conduct stakeholder mapping and commence a situation analysis. The first field trip ran for 11 days (Jul-Aug 2017) during which time the NF team, with support from a local third party (Vital Strategies), met face-to-face with diverse stakeholders (n=37) across the health ecosystem in São Paulo to discuss the initiative and continue to build knowledge about the people and context. Engagement efforts targeted cross-sectoral stakeholders including academics, hospital staff (primary care providers), politicians, philanthropic organizations, public health foundations, local NGOs and related healthcare groups (e.g., insurance).

Around the same time (Jun 2017), the NF team set up the first of what would become ongoing for rest of the initiative, regular (every three months) discussions and occasional workshops with City Hall.

During the second field trip, which ran for two weeks in Sept 2017, engagement efforts became more focused with particular emphasis given to the Secretary of Health of São Paulo, primary healthcare providers, local decision-makers, and stakeholders who carried good potential for further strategic partnerships (linked to the 5 strategic pillars of the initiative). In total, the NF team, with continued support from Vital Strategies, met with 24 people.

*Purpose of actions:*

On a practical level, the wide engagement conducted early on with stakeholders was intended to introduce the initiative and gather data to inform the situation analysis, as well as to identify support for it (local champions) and find a local implementation partner. Completion of the situation analysis provided more tangible contextual evidence on the state of services, system capacities and opportunities for multi-sectoral partnerships and informed a set of insights from which to reframe the form that the initiative should take for implementation. At a more personal level (cognitive and affective), targeted face-to-face engagement with stakeholders allowed for the voicing and discussion of opinions, which was expected to encourage a sense of validation for individual expertise and knowledge as well as potentially ignite a more open and creative problem-solving mentality when considering the possibilities of the initiative. Lastly, regular communications with key stakeholders were intended to provide status updates as well as promote ecosystem relationships and a growing connection/complementarity with the initiative.

*Justification:*

From early engagement work, it had become clear that there were only a handful of people and organizations that worked with the City Hall on health, primary care and prevention, and that these partnerships focused mostly on operational execution of healthcare delivery to provide care to the population. The Novartis Foundation perceived several opportunities to support the authorities in a needs-based and co-created way to focus on innovation, enhancing quality of care and brining international best-practices etc. This was a good indicator of new possibilities but also potential barriers and highlighted the need to be open to how the initiative might proceed but also remain true to the primary objective of building a multidisciplinary partnership upon which initial agreements had been formed.

Some relationships had been disrupted due to political changes at higher levels. The decision to put the entire focus on working very closely with the technical staff in the district and to co-create solutions at that level was to integrate the project to a point of being indispensable. This kept the project running and, in the end, proved fruitful for reestablishing the relationships that had been disrupted at higher levels as the outputs created (e.g., treatment protocols, training cycles for health workers, support to hypertension congress) were used to show proof of value-add.

Early stage and continued engagement efforts with lead decision makers are expected to encourage a sense of project ownership locally; consolidation of different local partnerships seeks to emphasize the importance of local reach to further promote buy-in and commitment as well as establish the appropriate levers to support implementation and future sustainability.

|  | **Activities** | **Actions** |
| --- | --- | --- |
| **6** | **Field Visit III (Part A): Tender process & preparatory meetings** | Request for proposals, final selection of implementation partner and formalization of governance committees. |
| **7** | **Field Visit III (Part B): Workshop with technical leaders (Governance)** | Kick-off Workshop with technical leads |

A third field visit lasting ten days was conducted in Dec 2017. Implementation partner candidates who had been shortlisted following a RfP published by NF in early November 2017, were invited to present their offer. Tellus were ultimately selected and time was spent negotiating a contract with them. A series of preparatory meetings were run with a view to formalize commitment from the Secretary of Health (signed ‘Letter of Intent’ agreement), and to set-up legal and governance support structures (i.e., steering and operational committees, impact evaluators, and other expert groups). Additionally, the search continued to identify additional local partnerships in line with the mission to build implementation capacity for multi-disciplinary work. This culminated in acquiring locally committed financial support for strategic pillars (i.e., Associacao Samaritano for community and prevention activities), cross-sectoral integration with public health programs that were already in place (i.e., Saúde na Escola, Agita São Paulo), and confirmed interest from academics to support impact evaluations.

Towards the end of the field visit, the NF team led a half-day kick-off workshop (Activity 7) with technical leads (n = 22 approx.) from City Hall (Itaquera district and East District), and the care provider (Santa Marcelina). The format incorporated informal (e.g., morning reception with coffee, brunch) and formal elements including a presentation and video about the initiative followed by open Q&A; a “world café” session with four round tables working to four different themes; final discussion with an open feedback followed by a closing session coupled with a ‘wish tree’. The implementation partner, Tellus, supported this workshop whilst their full contract was still under negotiation.

*Purpose of actions:*

The primary objectives were to **formalize commitments** to the initiative from all identified stakeholders and to **establish an operational standard** of shared decision making. The emphasis given to establishing formal **collaborative agreements** was intended to **promote transparency** around mutual engagements and commitments as well as act as an important step to **securing continuity** of engagement in shifting political times (e.g., election periods), which can often have destabilizing effects. Consideration of wider initiatives and partnerships was seen as critical to maintain control over the activities that would take place in the study area and build alignment with the initiative and other similar activities to improve the chances of the initiative being embraced by the system rather than rejected or isolated by it.

The workshop served to further promote **inclusivity and transparency** at the operational level. The mix of educational (i.e., presentation and video) and intensive participatory sessions (i.e., world café) followed by open discussion was designed to allow stakeholders to express and discuss their opinions and needs, and for implementation teams to continue to gather insights on what really matters. For example, during this process, stakeholders expressed strong opinions about the benefits of international vs local partners – the latter being preferred. This was recognized as a required feature of the initiative going forward but also validated the efforts already made to build local engagement and partnerships.

*Justification:*

Broad inclusion, with the intention of graduating stakeholder commitment, allows key stakeholders to contribute as system experts and engages them in critical and creative thinking about potential arrangements. Participatory activities are designed to allow for a shared affective and cognitive experience that helps to bring clarity to purpose and affirm a shared vision as well develop relationships.

|  | **Activities** | **Actions** |
| --- | --- | --- |
| **8** | **Project Kick-off** | **Project Kick-off - BHBC initiative with Tellus** |

The entire Tellus design team (n=8) spent 10 days preparing for a workshop to be led by them as the implementation partner in Feb 2018. Presentations about the demographics and cardiovascular disease burden in the study territory (Itaquera). Stakeholders (n=23) included: Government representatives from the Itaquera territory; East Zone coordination and Itaquera supervisors; the care provider partners (APS Santa Marcelina); the CDC as evaluation partner; and the Novartis Foundation.

*Purpose of actions:*

By bringing key operational stakeholders together face-to-face to actively engage in presentations and discussions, the workshop sought to promote collaborative working and to level expectations about the proposed project and reach agreement on how to move forward together, including the definition of specific roles and responsibilities (i.e., Tellus responsible for conducting the diagnosis process and partners responsible for sharing data, information and facilitating the process).

*Justification:*

The workshop space and format respectively allowed stakeholders to meet and build familiarity with each other and promoted a **transparent and** **inclusive approach to level expectations, foster ownership, and build consensus towards a shared vision** from which to move forward.

**DESIGN PHASE I: DIAGNOSIS**

|  | **Activities** | **Actions** |
| --- | --- | --- |
| **9** | **Desk research** | **Background research on disease, Better Hearts Better Cities initiative and local intervention context** |
| **10** | **CSD Matrix development** | **Consolidation of research findings into visual summary** |
| **11** | **Meetings with East Zone Coordination** | **Stakeholder validation of research tools & schedule** |

Throughout February, the whole design team (n=8) undertook an extensive in-depth needs assessment and embedded field research on several topics including the epidemiology of cardiovascular disease (global), experiences of the Better Hearts Better Cities initiative based on other participating cities (international), and the position of hypertension in terms of the São Paulo health system, including the patient community (Activity 9). Findings were then mapped into a multi-category CSD matrix (Certezas, Suposições e Dúvidas - specific design technique) to set out all hypotheses, certainties and doubts related to the challenges of the project. Based on this information, data collection tools and a field schedule were then drafted (Activity 10). The findings, tools and field schedule were subsequently presented to the core operational stakeholders (East Zone coordinator, Itaquera district supervisors, and the Care Provider, Santa Marcelina) (n = approx. 20) via face-to-face meetings (one day; design team split to cover different meetings) for validation and confirmation of next steps (Activity 11).

*Purpose of actions:*

Desk research was expected to **build familiarization** with hypertension and cardiovascular disease and the initiative content in general and then situate this knowledge in terms of the local setting and context (São Paulo city and Itaquera territory). The mapping of findings into the matrix allows for all data to be converted into a single visual resource. The creation of this visual evidence summary is a design method that is expected to serve several purposes: first, it enables the design team to see a target problem/situation from **different perspectives**; second, it acts as a **planning tool** (e.g., highlights where there are information gaps informing how interview and survey scripts should be designed); and third, to become an **educational and learning tool** to share and iterate with stakeholders (Activity 11). Face-to-face meetings are expected to continue to strengthen relationships.

*Justification:*

Conducting background research promotes an evidence-based approach to problem definition and is an expected standard for operational change within a health system. Transformation of data into a visual form creates a cognitive shift (innovation mindset) that can promote visibility of meaning and in turn, clearer understanding of issues and potential solutions. Lastly, open exchange of information and interpretations paired with shared decision-making, should lead to an improved **problem definition.**

|  | **Activities** | **Actions** |
| --- | --- | --- |
| **12** | **Interviews & co-creation sessions in Primary Care Clinics** | **Primary data collection - Interviews & co-creation sessions** |

Over the course of one month (Feb/March 2018), the design team (n=3) conducted 80 interviews with patients, supervisors and a diverse range of health professionals and support staff including: Community health agents (CHAs), nurses, pharmacists, technicians, physicians, clinic managers and administrators. Additionally, co-creation sessions (n=10) were conducted with patients and management councils.

*Purpose of actions:*

Interviews were designed to explore the proposed hypotheses and clarify doubts raised via the CSD matrix and to continue to build knowledge about specific **stakeholder** challenges, expectations and perspectives on hypertensive patients. By visiting interviewees in situ, the fieldwork was also an opportunity to gather new data about **care processes** and activities already taking place in health units as well as identify where other opportunities might lie. By exercising a deep dive into the details about people and processes, the team sought to **continually build knowledge** and allow for the **framing and reframing** of identified challenges to ultimately **reach a more precise position** on what the main focal areas of the initiative should be.

*Justification:*

A commitment to continuous learning about diverse stakeholders and system processes (i.e., whole system engagement) allows for deeper insights and a nimble response to new perspectives. This sensitivity builds empathy and in turn, better understanding, and is therefore more likely to support the development of a solution/innovation that is more powerful and more likely to be adopted (Beckman, 2007).

|  | **Activities** | **Actions** |
| --- | --- | --- |
| **13** | **Systematization, initiative status update & live event** | **Frame and reframe findings to establish a new innovation framework, stakeholder involvement & seizing real-time opportunities** |

A comprehensive analysis of all findings (recent interviews and existing evidence matrix) was conducted by the design team (n=3; 10 days) resulting in the development of a more complex framework, or ‘Systematization’, of assumptions and insights about the challenges to be addressed. In addition, a set of needs was interpreted for each of the target groups interviewed. All of these findings were presented to, and discussed, via a series of individual meetings (n=5) with high-level partners including the Novartis Foundation (n=2), City Hall (n=2), and the American Heart Association (n=2). By chance, a quick-win opportunity was taken to run an event on World Hypertension Day – sponsored by the World Hypertension Society – in a busy, mixed-use location (i.e., shopping mall, subway and train station) situated in the Itaquera territory. The estimated engagement impact of the communications materials and exchanges that took place during the event was around 100,000 people.

*Purpose of actions:*

The purpose of systematization was to create an **end-of-phase point of convergence** across all findings and to articulate a set of clearly **rationalized insights to confirm what the key challenges were and how these fit against the BHBC objectives.** The visual presentation of the results **formalized the learning** and put it into a more **accessible format** that could be readily shared with partners; as before, visual summaries are expected to support **collective understanding** of the findings and be used as tangible references to guide **shared decision-making** about next steps. Sharing findings for high level review is expected to promote active engagement on a shared platform and to **reaffirm partner relationships, responsibilities and roles** going forward into the next phase - ‘Exploration’.

*Justification:*

The systematization process is a design thinking technique and therefore integral to the approach. Use of this technique optimizes the **value of working with designers as it requires specialist skills** as well as creating an audit trail of process and evidence, which promotes external validity. Systematization also carries educational merit, helping to **streamline stakeholder learning and thinking and expedite action** within the innovation process. Keeping stakeholders informed via regular reviews continues to support **common agreement** on the project trajectory and reaffirms relationships.

**DESIGN PHASE II: EXPLORATION**

|  | **Activities** | **Actions** |
| --- | --- | --- |
| **14** | **Observational research: A day in the life of Community Health Agents (CHA)** | **Observational research - health professionals (primary) and patients (secondary)** |
| **15** | **Field research and open interviews with citizens** | **Field research - system structure and dynamics** |
| **16** | **Observational participation in activity groups held at Primary Health Clinics** | **Observational research – Hypertensive services in primary care** |

Multidisciplinary field research methods were embedded into the design thinking process during this phase. First, in March 2017, the design team (n=2) shadowed (observational research) CHAs (n=10) as they carried out home visits (n=100). Second, over the Mar-April period (6 days in total) the design team (n=2) conducted field research at each of the six pilot health units to assess services and infrastructure, as well as open interviews with unit staff (n=20) and hypertensive patients attending the clinic (n=10). The team also approached community citizens for interviews (n=10) via public spaces (e.g., one the street, subway stations, shops, and free markets). Third, design researchers (n=2) carried out further observational research (10 hours) at a primary care clinic where they observed an activity group for hypertensive patients and shadowed primary care clinic staff (health technicians, CHA, and managers).

*Purpose of actions:*

Field research promotes adherence to the principle of empathy, a central tenet of the design thinking approach. Through shadowing (Activity 14), designers seek to understand CHA perspectives on the scope of their own professional work and where they see room for improvement. Through firsthand experience designers are able to learn about the health care process of home visits and the daily life of HTN patients. Site visits and targeted interviews with the care provider and wider public are intended to build a more dynamic picture of the structural environment influencing the etiology of hypertension in the Itaquera territory, in particular, what it offers and how people interact with it in terms of their consumer habits and behaviors. Lastly, extended intimate observations are expected to establish a deeper understanding of current patient services and what motivates patients to engage with them. Altogether, these efforts added to the growing **evidence base** that will inform future action.

*Justification:*

Engaging with stakeholders through fieldwork is expected to show that there is a genuine interest in what they do and what they think, which helps to build trust between the research team and target subjects. Close working also helps to build empathy with stakeholders, and by seeking to capture a broader picture of the health environment through diverse perspectives, designers can be more critical in their thinking about needs and design goals. In turn, this deeper appreciation of lived experience is expected to enhance the evidence base for action and ultimately lead to better innovation.

|  | **Activities** | **Actions** |
| --- | --- | --- |
| **17** | **Co-creative meetings with managing councils of each clinic** | **Co-creation sessions** |

Individual managing councils are attached to each pilot site (UBS) and comprise healthcare professionals, health system managers and community representatives/clinic users. Tellus conducted six 1-hour meetings to cover each site. These meetings included a co-creative session.

*Purpose of actions:*

Managing councils represent the healthcare community – managers, healthcare professionals and patients – and are very important in the political sphere. Their needs and solution ideas were therefore considered essential to build the project and gaining their interest and support important to underpin the sustainability of activities to be undertaken within the initiative.

*Justification:*

As above for Activities 14-16

|  | **Activities** | **Actions** |
| --- | --- | --- |
| **18** | **Establishing baselines: Questionnaires & desk research** | **Collection of hard data at different levels of the health system** |

Also in April 2018, the design team (n=2) conducted interviews with clinic managers (n=6) and primary care health professionals (n=6) at each of the six pilot study clinics. One day was spent at each of the clinics (8 hours each day). During this time hard (e.g., prevalence of hypertensive patients, quantity of appointments) and behavioral data (e.g., use of protocols) were collected. Further contextual information was also gathered about the Itaquera territory and Brazilian health system. For Itaquera, quantitative data included socio-demographic characteristics, health services availability, and the location of food outlets, leisure and physical activity resources; qualitative data covered use of resources. At the national level, data were collected about the current use of care principles and guidelines, management types, infrastructure, IT systems, and the role of social organizations in the provision of care.

*Purpose of actions:*

Data collection was undertaken to establish a **baseline** to inform future evaluations and to extend the **ecological reach** of the CSD matrix with more contextual, structural and behavioral data about the health system and the community it serves. It was also another opportunity to reconnect with core stakeholders and **reaffirm developing relationships**.

*Justification:*

Baseline measures are necessary to enable future internal or independent evaluations. The use of the CSD matrix (systems tool?) recognizes the complex and adaptive nature of the health system and works to allow for the continuous inclusion and interpretation of up-to-date data. Repeated use helps to build a more detailed and coherent picture and lead to better innovation. Lastly, engaging with stakeholders repeatedly as experts helps to build empathy and relationships, process features that will influence implementation success.

|  | **Activities** | **Actions** |
| --- | --- | --- |
| **19** | **Evidence review, co-creative sessions and update - Patient adherence and health information** | **Global review of evidence on patient adherence & assessment of the local health information** |

In the same month (April 2018), the design team (n=2) conducted research on two new topics – patient adherence and local health information system (capacity and needs). For patient adherence, the team completed a review of the global and local (Brazilian) peer-reviewed literature on dimensions of patient adherence to hypertensive medications (e.g., knowledge, behavior). They also reflected critically on the theoretical underpinnings of behavioral change – specifically, the ‘Stages of Behavior Change’ model (Prochaska, 1997) – and how this applies to hypertensive patients. To investigate the functioning of the health information system, the team assessed the availability of data via the local routine health information register (e.g., adherence to hypertensive medications) as well as the system-wide health information needs. To understand more about the adherence challenges, pharmacists (n=6) from each health unit were interviewed and a further six co-creation sessions, with five patients in each, were run in each health unit.

*Purpose of actions:*

The literature review was intended to identify globally relevant **levers to support adherence** (e.g., the concept of ‘Triple adhesion’ – changing habits around medication, behavior and lifestyle). It also taught the design team more about the **theoretical** routes to behavior change and the progressive stages required. Interviews and co-creative sessions introduce current perspectives to round off topic knowledge so that any reflection on the status and story of local adherence data will be more complete and up-to-date and enable a more informed appreciation of how much change and support might be needed amongst the target population to meet health goals (e.g., long-term adherence). The assessment of the health information system was designed to get a read on the **quality of data** available and to **diagnose areas of need** to support adherence efforts (e.g., system requirements, faster access to data to inform decisions, simpler and more visual data presentation, synergistic data system connecting across actors to enable whole system activation, data categories, data centralization, and data form – digital versus other).

*Justification:*

These actions reflect the requisite **multidisciplinary approach** (i.e., psychological theories of change, evidence-based strategies and principles of design thinking such as empathy and user-focus) expected as part of the DT approach and initiative. This is considered necessary to best inform the problem definition and solution innovation process.

|  | **Activities** | **Actions** |
| --- | --- | --- |
| **20** | **Clinical guideline review vs real-world system functioning** | **Assess the reality of clinical guidelines use, professional training and user journeys using mixed methods (document review, meetings, and interviews) to inform the development of more practical guidelines.** |

During the same month (April 2018), the design team (n=2) ran meetings (n=10), incorporating co-creative sessions, with representatives (n=8) from different stakeholder groups including the NF, the Medical Society, Itaquera supervision and the care provider, Santa Marcelina. Questionnaires (n=18) were administered to health professionals (i.e., doctor, nurse, pharmacist, CHAs and other auxiliary staff) at UBS sites and interviews (n=18) were conducted with patients.

*Purpose of actions:*

The aim of these meetings was to identify and discuss the clinical guidelines available - there were three different versions - for the management of hypertension and current professional training curricula. The questionnaires helped to inform this process by showing how these guidelines are interpreted and executed in reality by diverse health professionals; what influences their use; and what training staff had received in relation to them. Interviews with patients were used to capture their user journeys within the health system. Altogether, these findings were to be used to identify gaps and possible reasons and solutions for them. Further, this direct and indirect research with diverse health system actors was expected to augment relationships through continued connection and sensitivity to user-perspectives.

*Justification:*

Working to illustrate experiences as a whole system of interactions can act as both an educational tool and critical evidence base to support innovation design. Taking the time to build stakeholder relationships throughout the course of the innovation process is seen as a fundamental ingredient to implementation success across multiple outcomes.

| **21** | **Checkpoints** | **Recurring meetings with Itaquera supervision throughout ‘Diagnosis’ and ‘Exploration’ phases (F2F)** |
| --- | --- | --- |

Throughout March-April 2018, the design team (n=2) scheduled fortnightly, face-to-face meetings with the technical leads (n=5) in the pilot area (i.e., Itaquera supervisors and supervision managers).

*Purpose of actions:*

These meetings acted as regular ‘checkpoints’ so that the different parties could work together on **logistics** (e.g., how and when to interview people) and to ascertain specific details about Itaquera training programs, UBS processes, and hypertensive treatment or similar. Regular contact was also a means through which stakeholders were kept up-to-date and everyday relationships promoted as the parties worked more closely together as an increasingly singular, but diverse, **project management and implementation team**.

*Justification:*

This process supported **shared decision making** which is expected to promote **deeper engagement** with the project and greater **commitment to roles and responsibilities**. It was anticipated that regular contact would keep the process moving and continue to build familiarity and a **sense of connection** between stakeholders.

|  | **Activities** | **Actions** |
| --- | --- | --- |
| **22** | **User journey formulation** | **Assemble data into visual summary - User journeys** |
| **23** | **Itaquera map** | **Assemble data into visual summary - Territorial map** |
| **24** | **Personas** | **Evidence-based creation of target 'Personas' for hypertension (descriptive)** |
| **25** | **Stakeholder map** | **Visualization of spheres of influence across health ecosystem** |
| **26** | **Characterization of health system and living environment** | **Visual profiles of pilot health units for intervention** |

Throughout April 2018, the design team internally created a series of evidence summary outputs based on all research findings to date. These included: ‘User journeys’ for healthcare workers (doctors, CHAs, pharmacists and managers) and patients in relation to hypertension (80 hours; 1 designer); an ‘Itaquera map’, representing the ‘urban plan’ of the hypertension ecosystem including demographic profiles, the location and coverage areas of UBS pilot sites, and the locations of public and private leisure and consumer facilities (e.g., shops and markets) (40+ hours; 1 designer). Third, descriptive ‘Personas’ depicting fictional Itaquera community members at risk of, or already living with, hypertension (40 hours; 2 designers). Fourth, a ‘Stakeholder map’, incorporating the entire Itaquera hypertension ecosystem (people and environment) (80 hours; 1 designer). And lastly, profiles of each pilot site were created to show their infrastructure and available services (80 hours; 2 designers).

*Purpose of actions:*

In order, these activities served to: show how the hypertension care process is experienced by each system user, and visually highlight the gaps and challenges that exist; provide a visual guide to help highlight different elements of the ecosystem that influence hypertension health status and behavior (e.g., shops and eating habits) and to work in tandem with other visual summaries (e.g., personas) to help identify target audiences and lifestyle change opportunities/agents; create a tangible and conceptually accessible summary of evidence that clearly defines the different target groups and their specific features and challenges as regards hypertension; visually summarize how the lives of hypertensive people, and those at risk, can be influenced by a range of system actors and indicate where this takes place across the health ecosystem (e.g., health services and local settings); and finally, to indicate the different types of services offered by the pilot sites and highlight areas of potential for solution design, particular challenges and to set expectations of the potential for change within practical parameters (e.g., resource allocation).

In the first instance, it was expected that each of these outputs would consolidate knowledge and understanding for the designers, and then take on additional roles as educational and co-creative tools to be used by stakeholders in the next phase – Cocreation – to enable efficient orientation to the nature of key problems and to guide/support solution generation.

*Justification:*

Visual tools draw on different applied disciplines that essentially explore how meaning is created and how meaning is communicated (e.g., communications and semiotics). Visual tools can translate complex information into something that is more intelligible to a wider audience and therefore more consumer-friendly. Seeing information in a different way also helps consumers to see problems from different perspectives, and in turn, derive different insights. In this way, visual formats serve to **break mental paradigms**, allowing solutions to be seen where one would not normally look for them.

Specific tools, like personas, are created for use throughout the design thinking process for several reasons. First, to understand specific challenges; second, to be used by workshops participants to springboard their initial ideas and then check the sense of them through repeat validation cycles; and third, to finally validate the solutions that have been created. Psychologically, working with personas helps to provide a clear and secure space for thinking, creating and reflecting.

|  | **Activities** | **Actions** |
| --- | --- | --- |
| **27** | **Development of design principles to guide design strategy** | **Set out design principles related to empathy, engagement and training to build and guide the co-creation strategy** |

Drawing on all the insights generated, the design team (n=2) worked internally (40 hours) to compile a definitive list of eight design principles to guide the design strategy.

*Purpose of actions:*

The creation of evidence-informed guiding principles is intended to serve the design team and all future co-creators as an innovation tool. In practice, designers used the principles to guide the development of an education training program for multi-professional primary care teams. The principles were also included in the materials to share with participants taking part in co-creation workshops (next phase). The overall purpose of the principles is to serve as a reference piece that stipulates the priority of keeping empathy and community engagement at the heart of the design strategy and to consistently promote connectivity with the target community and system when taking action or decisions.

*Justification:*

Innovation requires a shift in thinking and for many, this can be difficult. Having a tangible tool, like a set of principles, helps to promote a degree of psychological security by both enabling and anchoring creative thinking. Ideas and actions can be freely generated but also reflected back onto the principles to assess their suitability. In this way, all future activities can be anchored to an evidence-informed foundational platform to ensure that focus remains on key targets and that diverse activities can come together coherently.

|  | **Activities** | **Actions** |
| --- | --- | --- |
| **28** | **Development of guidelines for co-creation workshops** | **Build guidelines for co-creation workshops based on design principles** |

In March 2018, the design team (n=3) spent 40 hours developing guidelines for the forthcoming co-creation workshops (next phase). Visual summaries were used to organize learnings to date and format the workshop structure. Selected activities for the workshop centered on generating solutions to key problems and soliciting feedback on ideas, with an emphasis on engagement and empathy as set out in the design principles.

*Purpose of actions:*

The guidelines were created to act as an easy-to-digest summary of learnings that would be used to share **efficiently and effectively** with future workshop participants. By detailing the innovation process to date and distilling it into actionable next steps, it was anticipated that co-creators would be able to **engage in co-creation workshops** as equally knowledgeable **experts** and **primed to adopt a different mindset** to support successful co-creation – essentially take the baton and continue with the innovation process.

*Justification:*

Guidelines create a ‘comfortable ground’ (Beckman, 2007) from which co-creators can start to shift their mindset, learn and create through an empathetic lens and launch more fully into the next divergent design phase. The transparency and extensiveness of the process may help to promote stakeholder relationships and engagement. Guidelines also signify the closing of the ‘Exploration’ phase - the second point of convergence of the ‘double diamond’ design approach.

|  | **Activities** | **Actions** |
| --- | --- | --- |
| **29** | **Phase presentation to steering committee and broader group** | **End-of-phase presentation of all project findings for validation and definition of next steps** |

At the end of the ‘Exploration’ phase (April 2018), the design team (n=8) held a workshop split into two parts to conduct face-to-face meetings: first with the funder (two representatives), followed by other initiative partners, including district authorities, AHA, co-funders and others (n=10).

*Purpose of actions:*

The first meeting was a major checkpoint, to wrap up all the activities to date, validate them, and define next steps for the ‘Co-creation’ phase (e.g., the target challenges to focus on in co-creation workshops). Subsequent meetings were used to develop independent evaluation plans and confirm the roles of partners going forward. These meetings, along with others with diverse users, are call ‘validation rituals’ by designers, and allow the design process to move to the next set of activities.

*Justification:*

Meetings like this are also referred to as ‘validation rituals’ (Kursat Ozenc, 2017). Validation rituals have an important place in the design thinking process because they promote inclusivity and shared decision making amongst stakeholders which in turn endowers a sense of connectedness. Further, they help to ensure strategic alignment and build confidence in the approach before moving into the next set of activities.

**DESIGN PHASE III: CO-CREATION**

|  | **Activities** | **Actions** |
| --- | --- | --- |
| **30** | **Cocreation workshops with Primary Health Care clinic managers and healthcare workers (multidisciplinary)** | **Guided workshops to deep dive into problems, think about solutions, and prioritize the best** |
| **31** | **Cocreation workshops with hypertensive patients** | **Same as #30** |

**Activity 30** – In May 2018, the design team (n=4) conducted six, 2-hour co-creation workshops. Participants included clinic managers from each of the pilot unit sites (n=6), with one allocated to each workshop, and primary health care professionals extending to nurses, doctors, CHAs, pharmacists and other auxiliary staff (n= 40 approx.).

**Activity 31 -** Also in May 2018, the design team (n=4) ran four, 2-hour workshops with hypertensive patients (n=20 in total) living within the study territory (Itaquera). Patients were recruited from two public health activity groups being run at local primary health care clinics (study site units).

Workshops comprised three parts: First, supported by the visual summaries (i.e., user journeys, personas), a deep dive into the identified problems based on findings from the diagnosis and exploration phases. Second, using a selection of concept generation techniques (e.g., prototyping – low fidelity?), groupwork to generate as many solutions as possible to the problems presented. And third, using concept selection techniques (e.g., assessment against multiple variables such as resources need, impact, scaling impact and others), further groupwork to prioritize which of the generated solutions would be the best for clinics to adopt.

*Purpose of actions:*

The workshop format is essentially designed to bring people across the health system together – designers, stakeholders at the key operational unit level, and hypertensive patients – in order to create a **shared learning** experience/space. Within this, different stakeholders are recognized for their personal expertise and influence. For example, managers are responsible for engaging entire health units for intervention work, so it was important that they were present **(intervention champions**). Further, multidisciplinary teams may offer diverse perspectives on problems and solutions despite sharing a common workplace and hypertensive patients occupy an entirely new space altogether, further extending the bandwidth of perspectives. This inclusive approach is also intended to promote stakeholder relationships – old and new. Overall, the point is to maximize learning and engagement through diversity and active participation in the expectation that this will lead to better solution design and seed a sense of team building.

*Justification:*

The three-part structure helps to activate **creative/design cognition** (Cross, 2006), which allows participants to take new perspectives on problems and extends the range for solution generation. At the same time, having stakeholders, including designers, all working closely together and **actively engaged – mentally and physically** - helps to iteratively build relationships, engagement, understanding of purpose, ownership of the process and outputs, and possibly, influence stakeholder readiness to change. Lastly, through prototyping – that is, physically making proposed solutions – participant learning is promoted through experimentation (Thomke, 2003), which further influences thinking and overall engagement.

|  | **Activities** | **Actions** |
| --- | --- | --- |
| **32** | **Systematization of solutions** | **Collate ideas and solutions articulated by diverse end-users in co-creation workshops** |

After the co-creation workshops, three designers spent 40 hours collating all of solutions generated and added further details to them in terms of effort required, time required to develop, target group and other dimensions considered important to the overall strategy (e.g., impact on stages of the treatment cascade) and implementation parameters (e.g., minimum viable service required, ability to overcome bottlenecks, sustainability, and impact on disease burden and prevention.

*Purpose of actions:*

This process sought to improve the structure and definition of the ideas/solutions that had been generated so that they could be more easily assessed for prioritization.

*Justification:*

In co-creation workshops, the objective is to collect as many ideas as possible, but these are often unstructured and many are repeated. The process of clustering and detailing ideas helps on three main fronts: first, to group common ideas from which fewer, and more specific, solutions can be created; second, to create an audit trail from which solutions can be accessed again at any evolutionary point for further consideration or reworking; and third, to align and prioritize ideas according to the initiative and City Hall (government) goals and objectives.

|  | **Activities** | **Actions** |
| --- | --- | --- |
| **33** | **Solution discussion, prioritization and selection leading to the development of prototypes (high fidelity)** | **Solution matrix, final decision making, implementation plan & pilot prototypes** |

A series of meetings were held throughout June 2018, first with the initiative funder (NF) and then with local government (City Hall). These involved the design team (n=4) and representatives from the two stakeholder groups (n= 8). The refined solutions from the previous activity were plotted onto a matrix by relevance and resources needed for implementation and a final selection (n=30 solutions) was made by the group in line with overarching strategic goals (i.e., five pillars). **Budgets and timelines** were agreed, followed by a formal report to confirm the **implementation plan**. Afterwards, the design team worked to develop full prototypes (high fidelity) for the pilot implementation.

*Purpose of actions:*

The primary aim was to conduct a final review of the materials and reach agreement on what would be implemented in the final design phase – ‘Implementation’. The process of assessment and selection using the matrix was expected to provide a **comprehensive and transparent presentation** of all solutions so that they could be **fully considered and critically appraised** by the key stakeholders to reach **consensus on final decisions and planning**.

*Justification:*

This activity brings the high-level implementation team to another **end-of-phase point of convergence** (end of co-creation phase) and through shared decision making, builds in increasing **local responsibility and commitment**. Working directly with those who are informed about, responsible for and sensitive to, current and future resource availability, as well as other stakeholder needs, helps to ensure that the **contextual reality** is meaningfully considered during the selection process. In turn, this should help to promote the implementation of the solutions.

|  | **Activities** | **Actions** |
| --- | --- | --- |
| **34** | **Cocreation workshops with pharmacists** | **Guided workshops to deep dive into problems, think about solutions, and prioritize the best** |

In August 2018, building on earlier workshops with pharmacists working in Itaquera, the design team (n=2) ran additional co-creation workshops (n=4) with pharmacists working in primary care clinics (n=80) across all of São Paulo. Pharmaceutical representatives were recruited through the Pharmaceutical Coordination in the municipality of São Paulo, which represents each Regional Health Coordination of the Municipality of São Paulo. The workshops followed the same three-step structure as the co-creation workshops described above.

*Purpose of actions:*

Co-creation workshops create another opportunity to engage interactively with key stakeholders and extend knowledge about their priorities and opinions as to what would work best across the city-wide system as well as generate new solutions.

*Justification:*

Building on the positive relationships that had been developed through earlier work, the Pharmaceutical Coordination made a specific request to the initiative to explore opportunities to develop solutions for the whole city of São Paulo and not just the territory of Itaquera. This opportunity for extension also reflects an overarching objective for design thinking – to adapt and work to scale solutions.

# Bibliography

Beckman, S. &. (2007, Oct 01). Innovation as a Learning Process: Embedding Design Thinking. *California Management Review*, https://doi.org/10.2307%2F41166415.

Cross, N. (2006). *Designerly Ways of Knowing.* London: Springer-Verlag London Limited.

Kruk, M. G.-D. (2018, Sept 05). High-quality health systems in the Sustainable Development Goals era: time for a revolution. *The Lancet Global Health*, https://doi.org/10.1016/S2214-109X(18)30386-3.

Kursat Ozenc, F. a. (2017). Ritual Design: Crafting Team Rituals for Meaningful Organizational Change. In *Advances in intelligent Systems and Computing, Proceedings of the Applied Human Factors and Ergonomics International conference, 2017.* Springer Press.

Prochaska, J. a. (1997). The Transtheoretical Model of Health Behavior Change. *Am J Health Promotion, 12*(1), 38-48.

Thomke, S. (2003). *Experimentation Matters* (Vol. 1st ed.). Boston, MA.: Harvard Business School Publishing Corporation.
